# Construction and Effect Analysis of a Mixed *Actinomycete* Flora for Straw Returning to Albic Soil in Northeast China

**DOI:** 10.3390/microorganisms13020385

**Published:** 2025-02-10

**Authors:** Xiujie Gong, Yang Yu, Guoyi Lv, Yubo Hao, Lingli Wang, Juntao Ma, Yubo Jiang, Jiahe Zou, Jingyang Li, Qiuju Wang

**Affiliations:** 1 Heilongjiang Academy of Agricultural Sciences, No. 368 Xuefu Road, Nangang District, Harbin 150086, China; gxj2546@163.com (X.G.); yuyanghaas@163.com (Y.Y.); 13898181753@163.com (G.L.); yubohao2005@163.com (Y.H.); mmmjjjttt@sins.com (J.M.); vbojiang2007@163.com (Y.J.); zoujiahe1997@126.com (J.Z.); nkyljy@126.com (J.L.); 2 Shenyang Institute of Applied Ecology, Chinese Academy of Sciences, No. 72 Wenhua Road, Shenhe District, Shenyang 110016, China; wanglingli@iae.ac.cn

**Keywords:** albic soil, straw return, bacteria screening, actinomycetes, straw degradation

## Abstract

This research targets straw return in Farm 852’s albic soil, China. The soil is nutrient-poor with few microbes and slow straw decomposition. Through fixed-point sampling and bacterial screening, an actinomycete consortium consisting of four strains was assembled, and two of them were identified as new actinomycetes. After 7 days of fermentation, the lignocellulose degradation rates of this consortium outstripped those of single strains, with cellulose degraded at 69.07%, hemicellulose at 64.98%, and lignin at 68.95%. FTIR, XRD, and SEM verified the damage inflicted on the straw structure. Lab simulations found group D (with the consortium) had a higher straw weight loss rate than group C (with commercialized microbial agents) and controls. The compound actinomycetes stepped up the bacterial abundance with the passage of time. In contrast, their effect on fungal abundance was hardly noticeable, but they had markedly ameliorated the soil fertility. These findings prove that the microbial consortium effectively accelerates straw decomposition and boosts soil microbe abundance and fertility in albic soil. It shows great potential for straw return and provides a microbial solution for this field.

## 1. Introduction

Albic soil, confronted with notable low-productivity challenges and diverse obstacles [1,2], retains considerable importance in the realm of global grain production. In China, the total area of albic soil is approximately 5.96 million hectares [3]. In the Sanjiang Plain of Northeast China, one-quarter of the agricultural land is comprised of albic soil [4]. Albic soil is classified as low-yield soil due to the rapid depletion of organic matter content caused by its thin humus layer. These soils form under distinct climate: annual rainfall of 500–600 mm, cumulative temperature (≥10 °C) of 2000–2500 °C, freezing depth of 1–2 m, and 150–170 frozen days. Their parent material is chiefly Quaternary clay sediment. The groundwater table is 8–10 m deep [5]. In this context, wetting and drying cycles lead to the alternation of the oxidation–reduction process, resulting in the whitening of the sub-surface of the soil (a phenomenon known as leaching) [5]. High acidity and poor permeability are the main factors contributing to its low fertility (a condition termed soil degradation) [6]. Hence, improving low-yield albic soil holds significant strategic importance for ensuring food security. As previously reported, researchers have made strenuous efforts to rehabilitate albic soil and, consequently, enhance crop yield. Primarily, this has been achieved by increasing the concentrations of organic matter in the Ap horizon and the depth of available topsoil. For example, Albic soil has been remediated through the application of straw [7], biochar [8] and organic fertilizers [9], with the objective of elevating organic matter concentrations and facilitating the availability of more nutrients to plants. Furthermore, subsoiling and super subsoiling have been widely employed in agricultural production in albic soil areas [10], since they possess the capability to enhance soil nutrients and crop output [11].

Straw application is a commonly employed agronomic measure in developed countries. Conversely, in China, burning and discarding still account for a significant portion of straw disposal [12]. These improper agronomic practices have not only led to nutrient losses [13] but have also presented environmental challenges. For instance, the open burning of crop straw has become a major source of concern regarding air pollution [14]. In Northeast China, the annual output of corn straw is approximately 150 million tons. As a technical measure for safeguarding black soil in Northeast China, returning corn straw to the field is of utmost significance in terms of enhancing productivity, optimizing resource utilization, and protecting the environment. However, the elevated carbon-to-nitrogen (C/N) ratio in straw, coupled with the presence of polymers such as cellulose and lignin, substantially hampers its degradation process. In frigid regions such as northern China, the combined effects of high latitudes and climate change further impede straw decomposition [15,16,17]. Undecomposed straw not only disrupts crop germination and root development but also has a cascading effect on the yield and quality of subsequent crops [18]. Moreover, it furnishes an ideal milieu for the multiplication of pathogens and the survival of pest eggs. This scenario markedly heightens the risk of crop-related pests and diseases, including corn root rot, wheat sharp eyespot, and corn borer infestation [19,20,21]. In addition, due to the frigid climate of this region, the degradation of straw directly returned to the field is sluggish [22]. The one-year degradation rate is merely 45–60%, and the cumulative degradation rate in three years is only 80–90%. Hence, expediting the decomposition of straw is an effective means for returning straw to the field to boost soil fertility.

Microorganisms are essential for straw decomposition. Among them, actinomycetes stand out with their crucial role. Many studies have examined the changes in microbial communities during this process [23,24]. Research on lignocellulose-degrading microorganisms indicates that fungi are sensitive to environmental conditions and not ideal for large-scale production [25,26]. Bacteria have a relatively lower lignin degradation ability compared to fungi [27]. Actinomycetes, however, have a significant impact by successfully breaking the intractable bonds between the diverse components of straw and notably reducing the content of active substances in corn straw [28]. Moreover, although different microorganisms can produce lignocellulases, actinomycetes are the most advantageous producers as they not only generate abundant hydrolytic enzymes but also exhibit strong tolerance and adaptability to extreme environments such as acidic, alkaline, hypersaline, and hyperthermal ones [29,30,31]. Therefore, bioprospecting for novel actinomycetes taxa is important for industrial and agricultural production.

This study aims to tackle issues such as sluggish straw decomposition and undernourished albic soil during the straw returning process in the albic soil area of Sanjiang Plain, China (Farm 852). Through screening actinomycetes possessing straw decomposition potential and employing methods like culture identification and analyses of straw decomposition ability, an actinomycete consortium suitable for straw returning in albic soil was constructed and refined. By simulating the field albic soil environment in the laboratory, the straw decomposition ability of the specially prepared actinomycete consortium was analyzed, and the impact on the main nutrient components of the soil was examined. This work offers an actinomycete consortium scheme for enhancing the effect of straw returning in albic soil.

## 2. Materials and Methods

### 2.1. Characteristics of Albic Soil and Corn Straw

Albic soil samples were collected from the albic soil in the typical area of Farm 852 (132°28′ E, 46°26′ N) in Heilongjiang Province, China, in April 2023. A random five-point sampling method was adopted. The collected samples were then mixed into a single composite sample. Three replicate samples were randomly taken. The samples were collected at depths of 0–20 cm (Ap—topsoil layer) and 20–40 cm (Aw—albic soil layer), respectively [32]. The corn straw used in the experiment was collected from the Science and Technology Park of Heilongjiang Academy of Agricultural Sciences after the harvest in autumn 2023.

### 2.2. Isolation and Characterization of Lignocellulose-Degrading Actinomycetes

To obtain in situ strains having the ability to decompose corn straw in alpine regions characterized by Albic soil, three soil sample collection sites were designated in this study: Sample 1 was collected from the albic horizons (Aw) in albic soil area of Farm 852 in October, 2022; soil Sample 2 was collected from the straw pile in albic soil area of Farm 852 in April 2022; and soil Sample 3 was collected from the straw pile of Mohe (121°12′ E, 52°10′ N) in April 2023, in Heilongjiang Province, Northeast China. In each region, 5 sampling sites were selected. From each of these sites, 5 points were randomly chosen, and the samples were combined for processing. The soil sample was naturally air-dried in a shaded location. After being ground into fine powder within a mortar, it was placed in a triangular flask. An appropriate quantity of sterilized glass beads was added. Subsequently, the mixture was agitated in an air oscillator operating at 180 rpm for 0.5 h. Then, it was diluted and spread on the surface of ISP2 medium for separation. The ISP2 medium was composed of 0.4% glucose, 1% malt extract, 0.4% yeast extract, 2% agar, and had a pH range from 7.0 to 7.4. The culture was incubated in an inverted position in a 28 °C incubator.

The above-mentioned strains were pure-cultured, sub-cultured for 3–5 generations, and stained with Congo red [33]. Strains with larger transparent circles were selected for subsequent experiments. In the filter-paper disintegration experiment, performed according to the relevant literature methods [34], strains capable of reducing the filter paper to a pulpy consistency were designated as “+++”. Strains that exhibited a certain degree of degradation effect on the filter paper were labeled as “++”, while those that caused no obvious damage to the filter paper were marked as “+”.

### 2.3. Molecular Biological Identification of Lignocellulosic Degrading Actinomycetes

Reference methods for molecular biological identification: in ISP2 medium, the cell biomass for DNA extraction was obtained by culturing at 28 °C. Standard procedures were employed to extract genomic DNA and amplify the 16S rRNA gene by PCR. The PCR products were purified, cloned into vector pMD18-T (Takara, Dalian, China), and sequenced using the Applied Biosystems DNA sequencer (Model 3730XL). The complete 16S rRNA gene sequence was obtained and compared with several sequences in the GenBank/EMBL/DDBJ database.

The determination of phylogenetic neighbors and similarity analysis was achieved using the EzBioCloud server [35]. Multiple alignments of the 16S rRNA sequences of the tested strains were conducted with the representative sequences of closely related organisms in the genus Streptomyces obtained from the GenBank/EMBL/DDBJ databases using Clustal X 1.83 software. Phylogenetic trees were generated with the neighbor-joining [36] algorithms using the Molecular Evolutionary Genetics Analysis (MEGA) software, version 7.0. Bootstrap analysis was carried out based on 1000 resampling to ensure the stability of the resulting tree topologies [37].

### 2.4. Construction of Actinomycetes Community

To validate the adaptability of strains originating from diverse sources (non-albic soil samples) within the albic soil environment, strain *Streptomyces* sp. NC5^T^ (patented strain number: GDMCC 63431) was isolated from the soil beneath the Heihe soil straw stack in 2021, exhibiting superior cellulose degradation performance. The propagation of the cellulose-degrading strain *Streptomyces* sp. NC5^T^ in the soil was ascertained through a colonization test. The characteristics of strain *Streptomyces* sp. NC5^T^ and the experiments on colonization of albic soil are shown in Figure S1.

For the purpose of constructing a compound microbial community, a confrontation culture was initially conducted on diverse strains possessing lignocellulose degradation capabilities. Two distinct purified single strains were streaked onto ISP2 solid medium and then incubated at 30 °C for a period of five days. Subsequently, the growth status of the colonies was observed.

To determine the ability of lignocellulose-degrading actinomycetes, active strains were analyzed for their degradation ability in terms of enzyme activity and degradation of straw’s lignocellulosic components. Enzyme activity indicators included FDA (filter paper activity), cellulase (endoglucanase, exoglucanase, and β-1,4-glucanase), hemicellulase (xylanase), and ligninase (laccase). The determination methods refer to our previous studies [28,38]. The production of 1 μg of glucose per minute is defined as one unit of enzyme activity. The determination of cellulose, hemicellulose, and lignin in corn straw refers to the VanSoest washing method [39].

In light of the experimental outcomes presented above, specific actinomycetes were meticulously selected and assembled to evaluate their enzymatic activity and straw degradation proficiency.

### 2.5. Decomposition Effect of Specific Actinomycetes on Corn Stalk

The straw was obtained from corn stalks. It was first flattened and then cut into segments with a length of 1 cm. After that, it was dried for further applications. The culture medium for mixed actinomycetes was composed of 0.3% yeast extract, 0.3% dipotassium hydrogen phosphate, 0.03% magnesium sulfate, 0.01% ferrous sulfate, 0.04% zinc sulfate, 0.01% calcium chloride, and 15 μL Tween 20. Using corn straw as the sole carbon source, the formulation of the IPS2 liquid medium was optimized as follows: 2% (*w*/*v*) corn straw and 0.4% (*w*/*v*) yeast extract, while maintaining the pH within the range of 7.0. The cultivation was carried out in shake flasks at 30 °C for 7 days, with an inoculation amount of 3% (*w*/*w*) of dry matter. For each sampling time point, three flasks were randomly selected for subsequent analyses.

Fourier Transform Infrared Spectroscopy (FTIR, Instrument Models: Thermo Nicolet IS5/IS10) analysis was conducted to explore the structural and compositional alterations of corn straw samples following degradation by different experimental groups. Specifically, 1 mg of corn straw was meticulously pulverized in combination with 400 mg of KBr and then compressed into pellets. The scanning wavelength ranged from 4000 to 400 cm^−1^. An X-ray diffractometer (XRD, Instrument Model: Bruker D8, Standard Number: JY/T 0587-2020, Bruker AXS, Karlsruhe, Germany) was utilized to record the crystallinity of the straw samples. The angular range was set from 5 to 90° (2θ) with stepwise scanning. The radiation source was Nickel-filtered Cu-K (λ = 0.15417 nm), and the scanning conditions were a voltage of 40 kV and a current of 30 mA. The crystallinity index (CrI) of the Residual Straw Fiber (RSF) was calculated in accordance with the Segal empirical method [40,41]. An XRD test was commissioned to Shanghai Weipu Testing Technology Group Co., Ltd., Shanghai, China (https://www.weipugroup.com/, accessed on 26 January 2025). The Hitachi - manufactured S - 3400 - N Scanning Electron Microscope (SEM) (Hitachi High-Tech, Tokyo, Japan) with a 5 - volt acceleration voltage was utilized for the morphological characterization of the materials. The samples were examined under an acceleration voltage of 5 kV, and 1000× images were obtained to precisely depict the structural and interfacial modifications of the corn straw samples.

### 2.6. Laboratory Simulation: The Influence of Actinomycetes on Albic Soil with Straw Returned

#### 2.6.1. Experimental Design

To precisely analyze the impacts of specially formulated mixed actinomycetes on albic soil under the circumstance of straw return, four distinct treatment plans were carefully devised via laboratory simulation experiments, detailed as follows:

Treatment A: “Aw soil”. The soil was dried and sieved through a 40-mesh sieve.

Treatment B: “Aw soil + straw”. Using the same soil as in Treatment A, the straw was dried, pulverized, and then sieved through a 40-mesh screen.

Treatment C: “Aw soil + straw + Woobao”. It combined pretreated white soil and straw. Woobao, a powdered commercial lignocellulose decomposition agent, was applied at 2 kg per mu. Given a straw application rate of 800 kg per mu, 0.25 g of Woobao was added per 100 g of straw.

Treatment D: “Aw soil + straw + actinomycetes (inoculation amount: 3% of dry matter)”. Using the same Aw soil and straw as previous treatments, after crushing corn straw, 60 g of Aw soil and 1.8 g of straw were placed in each 250 mL conical flask, with the water content adjusted to 60%.

Controls were established, including a straw-free control (Treatment A) and a straw-degrading-bacteria-free control (Treatment B). By comparing the degradation effect of commercial straw decomposition agents, the degradation capacity of the specially prepared actinomycete flora could be more intuitively observed. Sampling was done at weeks 2, 4, 6, 8, 10, 12, and 14 of cultivation. Then, the samples were put into sterile sealed bags for index determination.

#### 2.6.2. Analysis of Soil Chemical Properties

To verify the impact of the addition of exogenous microorganisms on the chemical properties of the soil during the process of straw return to the field, the primary testing indices focused on were soil organic matter (SOM), total nitrogen (TN), total phosphorus (TP), total potassium (TK), and pH. Various soil samples were subjected to measurement, and the specific measurement methodologies were as follows: For SOM, refer to “Soil Agrochemical Analysis (Third Edition)”, edited by Bao Shidan, published by China Agriculture Press in 2000, pages 34–35 (the method of potassium. For the determination of Total Nitrogen (TN), the LY/T 1228-2015 standard, which was specifically designed for the Determination of Nitrogen in Forest Soils, was adopted and implemented. Regarding TP and TK, the HJ 832-2017 standard (Soil and Sediment Total Metal Element Digestion by Microwave Digestion and ICP Determination) was adopted. For pH, the NY/T 1121.2-2006 Soil Testing Part 2: Determination of Soil pH was used. The ambient temperature during the test was 21.5 °C, and the relative humidity was 35%. All the test work was commissioned to the National Energy R&D Center for Biomass (NECB) of China Agricultural University.

#### 2.6.3. Microbial Diversity Analysis

The microbial diversity analysis was performed using the Illumina HiSeq sequencing platform and the Paired-End approach to build small-fragment libraries for sequencing. Via filtering, clustering or denoising, species annotation, and abundance analysis of Reads, the species composition of the samples was disclosed [42,43]. This task was accomplished by Shanghai Majorbio Bio-Pharm Technology Co., Ltd. in Shanghai, China, through Majorbio Cloud (https://cloud.majorbio.com/, accessed on 26 January 2025).

### 2.7. Statistic Analysis

In the previously described experiments, three replicate samples were set up, and the mean was calculated. The mean’s standard error (±) was computed via three repeated measurements using Microsoft Office Excel 2010, and the mean values of treatment groups were compared at *p* < 0.05 using the LSD method. Data plotting was done with Origin 2021.

## 3. Results and Discussion

### 3.1. Characteristics of Albic Soil Samples and Corn Straw

Based on the determination of relevant indicators in different soil layers of the albic soil area in Farm 852 of the Sanjiang Plain, the results are shown in Table 1. All indicators in the albic horizon were relatively low. The total organic matter (SOM) content ranged from 9.34 to 15.32 g/kg, the TN content was between 0.69 and 1.01 g/g, the TP content was from 0.29 to 0.35 mg/g, and the TK content was within 16.20–17.36 mg/g, all of which were lower than those in the black soil layer. The typical poor chemical properties of albic soil have also been confirmed in soil samples from other regions [44]. This suggests that the Aw soil exhibits a state of nutrient depletion and insufficiency, creating an adverse environment that severely restricts crop growth and development. Given this situation, the remediation and enhancement of the nutritional profile within the albic horizon of the albic soil becomes an imperative and crucial task. Through comprehensive soil improvement measures, such as precise nutrient supplementation, organic matter addition, and soil structure optimization, it is possible to rectify the nutrient imbalance, enhance soil fertility, and ultimately create a more hospitable substrate for sustainable crop production, which is vital for ensuring agricultural productivity and food security.

The main components of straw had the following contents: cellulose at 39.7%, hemicellulose at 29.1%, lignin at 8.6%, and other matters at 22.6%.

### 3.2. Screening of Actinomycetes with Lignin-Degrading Ability of Straw

#### 3.2.1. Preliminary Screening of Active Actinomycetes

A total of 383 single actinomycetes colonies were isolated from the soil samples. Specifically, 76 colonies were screened from Sample 1, 204 from Sample 2, and 103 from Sample 3. The naming convention was as follows: the letters such as A, NC, and MD denoted the names of the screening media, while the numbers 3, 4, and 5 corresponded to the dilution multiples of the soil sample coating, namely 10^−3^, 10^−4^, and 10^−5^, respectively. The selected strains were numbered in sequence and then transferred to the oatmeal Agar medium. These strains were incubated at 28 °C for 15 days. Subsequently, the morphology of the single colonies was photographed and counted. It was found that there were 84 strains possessing lignocellulose degradation ability. The ratio of the diameter of the transparent circle to that of the colony for the 84 active strains with cellulose degrading ability was calculated, as presented in Table S1.

#### 3.2.2. Disintegrating Ability of Filter Paper Strip

For the purpose of assessing the secretion of cellulase, 17 strains with a ratio of transparent circle diameter to colony diameter exceeding 3.93, along with a novel strain *Streptomyces* sp. NC5^T^ (patent strain number: GDMCC No. 63641), were selected for filter strip decomposition analysis. The outcomes of filter paper decomposition are presented in Table 2. The strain numbers CP82, MD15, MD31, MD50, MD55, MD63, MD68, JD30, NC5, JSTG24, and GS9 exhibited excellent performance (+++), where the filter paper lost its original shape and was nearly disintegrated into a pulpy consistency. Strain numbers SY6, MD43, and MD53 demonstrated a certain degree of degradation effect on the filter paper (+ +), yet the degradation was restricted, with the filter paper merely being bent rather than reaching a pulpy state. Strain numbers MD7, MDB7, MD44, and MD28 were capable of degrading (+), but the degradation efficacy was relatively suboptimal (Figure S2). The strains with outstanding performance (+++) were designated for subsequent experiments.

#### 3.2.3. Enzyme Activity of the Strains

**Figure 1:**
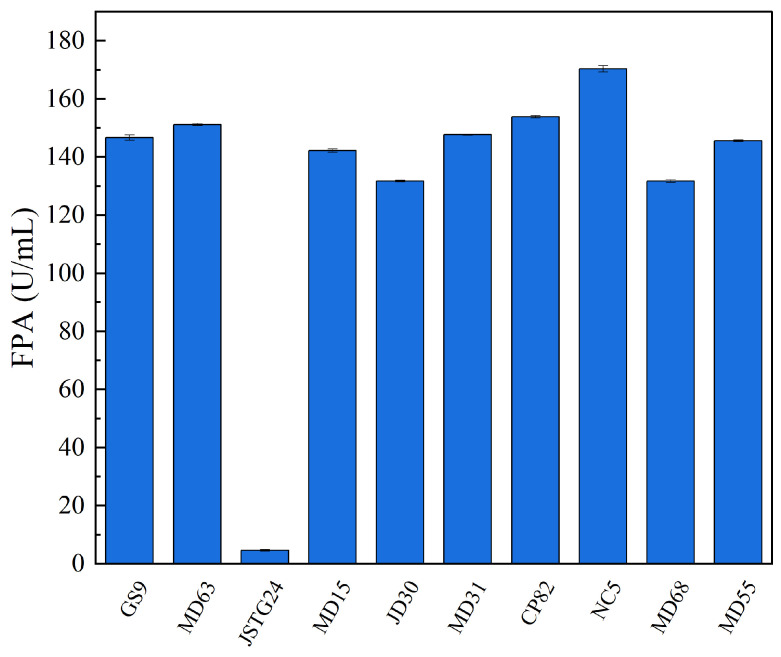
The results of FPA activity of each strain. Error bars are the standard error (n = 3).

**Figure 2:**
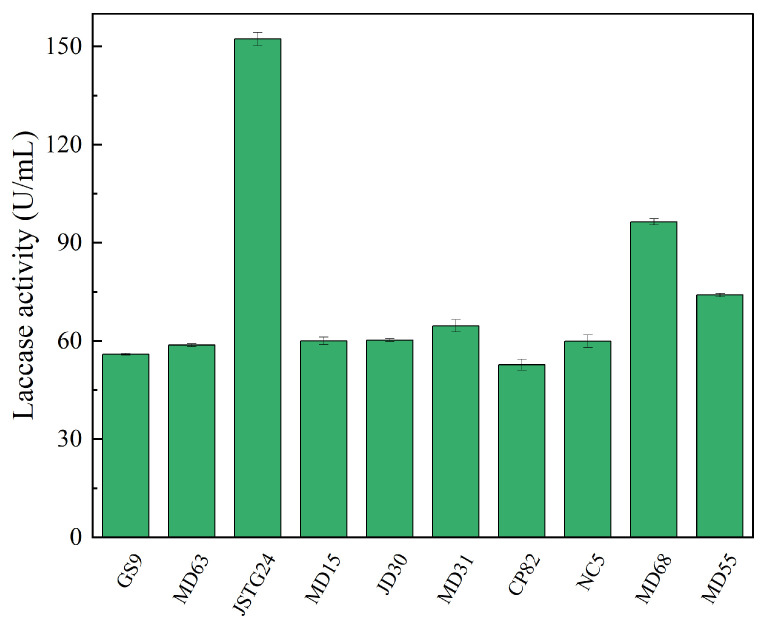
The results of lignin degrading ability (laccase) of each strain. Error bars are the standard error (n = 3).

**Figure 3:**
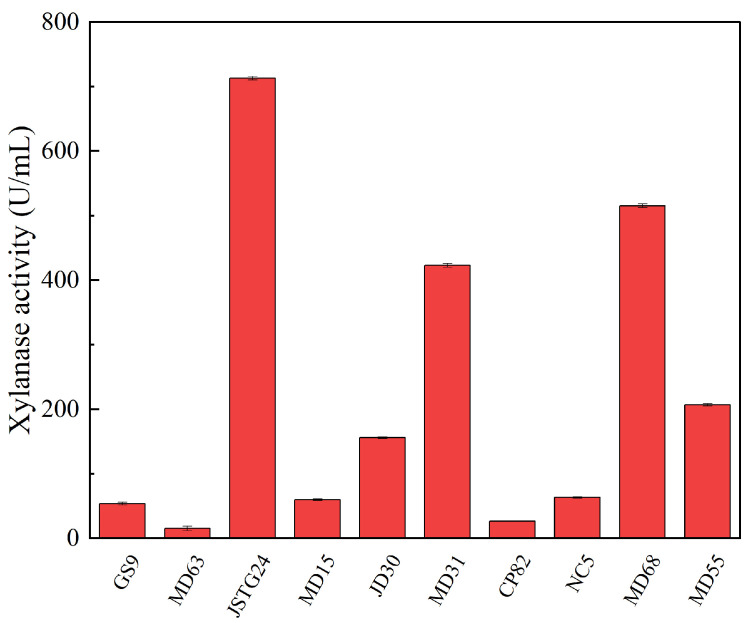
The results of hemicellulose degrading ability (xylanase) of each strain. Error bars are the standard error (n = 3).

**Figure 4:**
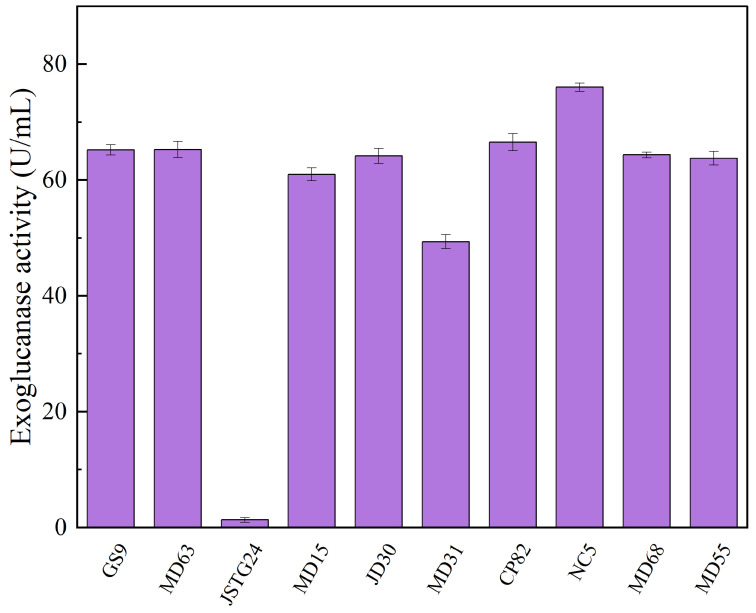
The results of cellulose degrading ability (exoglucanase) of each strain. Error bars are the standard error (n = 3).

**Figure 5:**
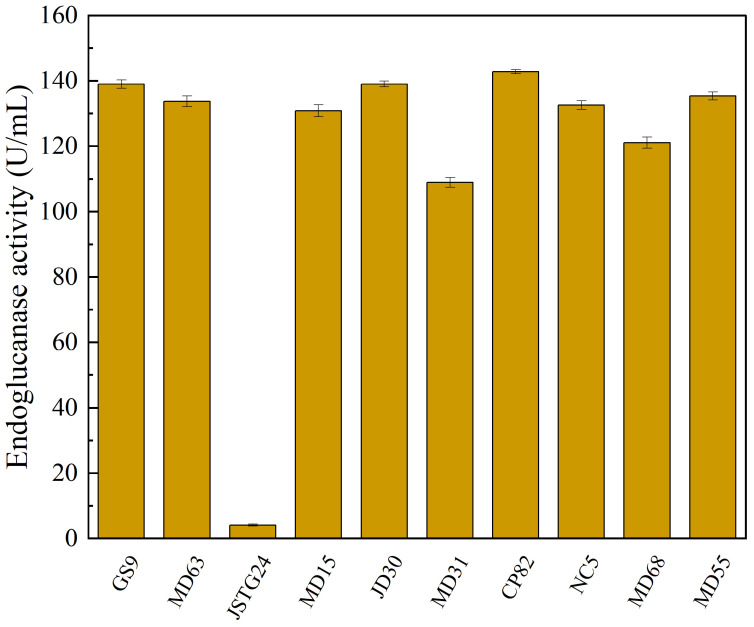
The results of cellulose degrading ability (endoglucanase) of each strain. Error bars are the standard error (n = 3).

**Figure 6:**
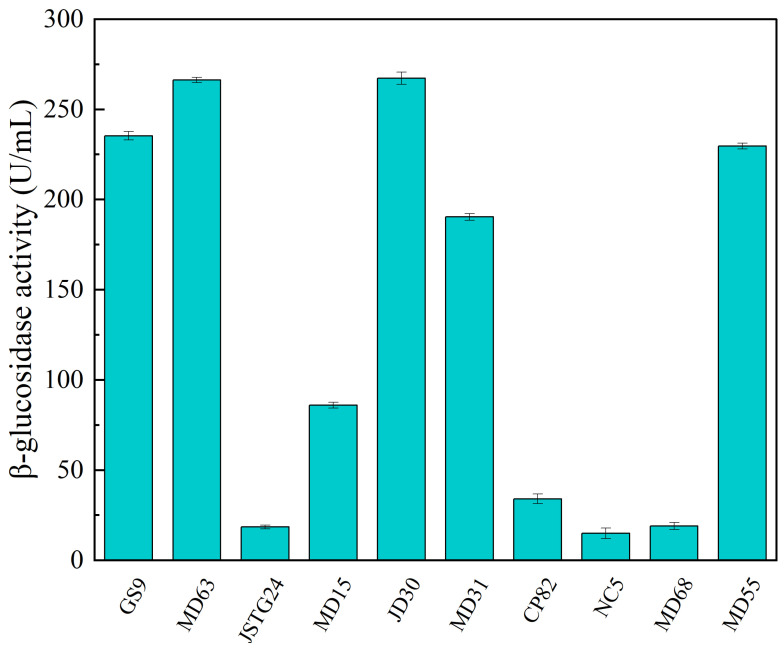
The results of cellulose degrading ability (β-glucosidase) of each strain. Error bars are the standard error (n = 3).

Microbial degradation of straw principally transpires through enzymatic exudation [45]. The strains exhibiting the maximal filter paper activity (FPA) were NC5, CP82, JD30, and MD63, with their corresponding enzymatic efficacies reaching 172.74, 153.77, 151.67, and 151.16 U/mL, respectively (Figure 1). The strains JSTG24, MD68, and MD55 evinced the supreme laccase activity, with their enzymatic intensities being 152.30, 96.40, and 74.00 U/mL, respectively (Figure 2). With regard to xylanase activity, the strains JSTG24, MD68, and MD31 manifested the zenith values, with enzymatic potencies of 712.74, 515.09, and 422.84 U/mL, respectively (Figure 3). NC5, CP82, MD63, and GS9 exhibited the foremost exoglucanase activity, with enzymatic magnitudes of 76.02, 66.52, 65.25, and 65.18 U/mL, respectively (Figure 4). CP82, JD30, GS9, and MD55 harbored the preponderant endoglucanase activity, and their enzymatic intensities were 142.76, 138.99, 138.98, and 135.33 U/mL, respectively (Figure 5). JD30, MD63, GS9, and MD55 demonstrated the preeminent activity of β-1,4-glucosidase, with enzymatic capacities of 267.24, 266.26, 235.28, and 229.58 U/mL, respectively (Figure 6).

#### 3.2.4. Changes in Straw Component Degradation by Different Strains

Corn straw is principally constituted by cellulose, hemicellulose, and lignin. The degradation capabilities of diverse strains with respect to the distinct constituent elements of the straw were explored. In accordance with the experimental outcomes, the strains MD63, NC5, and MD68 exhibited the highest cellulose degradation capacity, with cellulose degradation rates amounting to 63.4%, 62.97%, and 61.01%, respectively. The strains JSTG24, MD68, and MD31 manifested the highest degradation rates of hemicellulose, which were 55.36%, 54.54%, and 48.35%, respectively. Regarding the degradation rates of lignin, they were 71.16% for JSTG24, 59.88% for MD68, and 50.35% for MD55, respectively (Table 3).

#### 3.2.5. Molecular Biological Identification of Strains

Based on the experimental results regarding enzyme activity and straw degradation ability as described above, a total of 10 strains with relatively high lignocellulosic degradation ability were isolated and identified. Molecular biological identification was carried out on these 10 active strains. The specific details are presented as follows: the CP82 strain belonged to *Nonomuraea zeae*; the JD30 strain was affiliated with *Streptomyces broussonetiae*; the MD31 strain belonged to *Streptomvces canus*; the MD55 strain belonged to *Streptomyces fagopyri*; the MD63 strain belonged to *Streptomyces cinnabarigriseus*; the MD15 strain belonged to Streptomyces *phaeochromogenes*; the JNC5 strain belonged to *Streptomvces malavsiense*; the JSTG24 strain belonged to *Streptomvces barringtoniae*; the GS9 strain belonged to *Streptomyces rishiriensis*; the MD68 strain belonged to *Pseudomonas migulae*. The detailed content regarding the sequencing results of the active strains is presented in Table 4.

Fortunately, following the identification process, it has been clearly established that the strain labeled CP82 is a newly discovered strain, and its taxonomic classification falls under *Nonomuraea* sp. The preservation protocol for this novel strain has been successfully concluded, and it has been assigned the preservation numbers GDMMCC 4.350 and JMC36661. The phylogenetic tree and colony morphology of the *Nonomuraea* sp. CP82^T^ strain can be referred to in Figure S3.

### 3.3. Construction of Mixed Actinomycetes for Degrading Lignocellulose

#### 3.3.1. Confrontation Test

There are interactions such as nutrient competition, antagonism, or inhibition among different microbial strains [46]. To confirm the antagonistic effects among the strains, a confrontation test was conducted on the 11 strains with relatively good cellulose degradation effects screened in this study. The results are shown in Table S2. It can be seen that some strains exhibited antagonistic effects, such as NC5 and JSTG24. Notably, MD50, with slow growth, was excluded from compound microbial inoculum candidates. Based on the experimental results aforementioned, the microbial community was composed of NC5 and CP82 (two novel actinomycetes), along with JD30 and MD68 (low-temperature active degrading bacteria, whose detailed experimental data have not been provided in this study). NC5, CP82, and JD30 were, respectively, the strains that displayed the highest cellulolytic enzyme activities. In contrast, MD68 was a strain with relatively high activities of hemicellulase and ligninase (As shown in Figure 2 and Figure 3, respectively). The prominent characteristic of this compound microbial community was that it incorporated the strains with the optimal cellulose degradation capabilities. Moreover, the enzyme activities among the strains within the community were capable of mutually complementing each other. Different bacterial strains within this microbial community possessed diverse features. The rare actinomycetes NC5 and CP82 had specific straw-degrading abilities, and the low-temperature degrading bacteria JD30 and MD68 were able to participate in the degradation process under specific environmental conditions.

#### 3.3.2. Analysis of Degradation Effect of Straw with the Mixed Actinomycetes

In accordance with the results presented in Table 3, the corresponding degradation rates of cellulose, hemicellulose, and lignin by the mixed actinomycetes were 69.07%, 64.98%, and 68.95%, respectively. The capacity of the mixed actinomycetes in degrading cellulose were manifestly superior to those of the individual strains, namely MD63, NC5, and MD68, which, respectively, displayed cellulose degradation rates of 63.4%, 62.97%, and 61.01%. In the context of hemicellulose degradation, the mixed actinomycetes exhibited a considerably enhanced capacity and competence as opposed to the single strains JSTG24, MD68, and MD31, with degradation rates of 55.36%, 54.54%, and 48.35%, respectively. Nevertheless, in the sphere of lignin degradation, although the mixed actinomycetes showed a relatively attenuated degradation capacity in comparison to JSTG24 (exhibiting a degradation rate of 71.16%), they still held a preponderant position over the other remaining strains.

**Figure 7:**
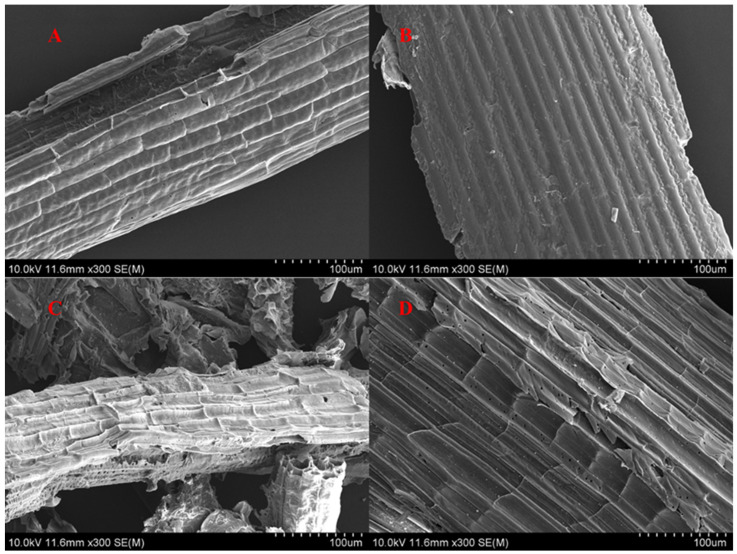
Corn stalk morphologies in pre- and post-treatment states. (**A**,**B**) signify the untreated straw (CK), and (**C**,**D**) represent the microbial-treated experimental group.

The experimental outcomes unequivocally validated that the mixed actinomycetes possessed a decidedly prominent lignocellulose degradation potential when contrasted with the single strain, thereby highlighting and underscoring the distinct and remarkable advantage of the mixed form in the process of lignocellulose disintegration and decomposition. Employing scanning electron microscopy (SEM) to characterize the straw architecture enabled direct apprehension of the microstructural modifications within the straw under disparate treatment regimens. CK refers to the untreated corn straw. As shown in Figure 7A,B, its surface was smooth, dense and gapless, with a waxy layer. This compact surface impedes the degradation process of cellulose [47]. In the experimental group (Figure 7C,D), the surface was rough, the gaps enlarged into pores, and the internal structure was irregular and had many small voids, which was distinctly different from the control. Based on the observation, the compound actinomycetes consortium was capable of disrupting the outer surface and exerting an effect on the internal structure of the straw, thereby actively facilitating the degradation of the straw.

**Figure 8:**
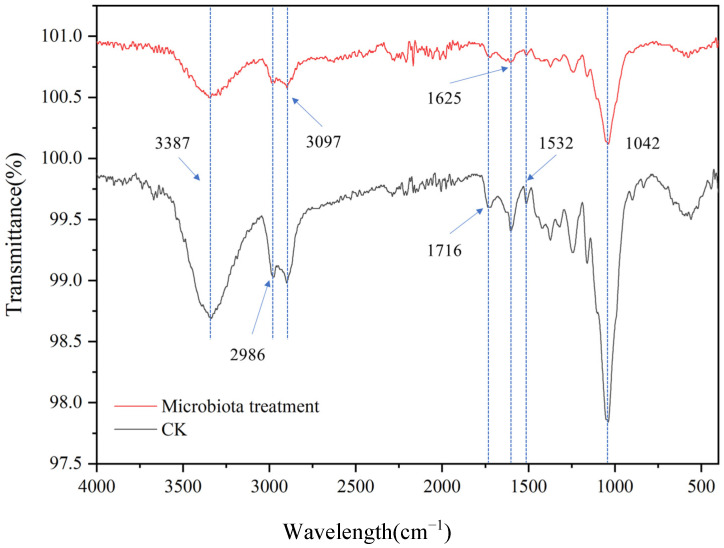
FTIR analysis. CK represents the untreated straw; microbiota treatment refers to the straw samples treated with the mixed actinomycetes flora.

In order to thoroughly understand the impact of microbial communities on the structure and properties of corn straw, we utilized Fourier Transform Infrared Spectroscopy (FTIR) to analyze the chemical changes within the samples of corn straw. Typically, the characteristic absorption peaks of cellulose emerge in regions like approximately 1000–1200 cm^−1^, 1300–1400 cm^−1^, and 1600–1700 cm^−1^ [48]. As illustrated in Figure 8, the absorption peak at 1042 cm^−1^ corresponded to the asymmetric stretching vibration of the C-O-C bond in cellulose, while the peak close to 1370 cm^−1^ was associated with the C-H bending vibration of cellulose. The peaks at 3387 cm^−1^ and 1716 cm^−1^, respectively, corresponded to the O-H stretching vibration and the C=O stretching vibration in cellulose. The absorption peak positions of hemicellulose showed a certain degree of overlap with those of cellulose; nevertheless, it also had its own distinctive characteristic peaks. For instance, the absorption peak around 1716 cm^−1^ could be ascribed to the C=O stretching vibration of acetyl groups and uronic acids in hemicellulose. The characteristic absorption peaks of lignin in the FTIR spectrum were relatively intricate and mainly appeared in regions such as 1500–1600 cm^−1^, 1400–1500 cm^−1^, and 1200–1300 cm^−1^. Among them, the peaks near 1532 cm^−1^ and 1625 cm^−1^, respectively, corresponded to the benzene ring vibration and the C=O stretching vibration in lignin [49]. By virtue of the degradation data of straw components, the impact of mixed actinomycetes on straw could also be substantiated (Table 3). Compared with the control group, the sharpness of each peak was lower, indicating that the content of cellulose, hemicellulose, and lignin in the straw of the experimental group (microbiome treatment) was reduced [50]. The results showed that the addition of microbial agent promoted the degradation of straw components.

**Figure 9:**
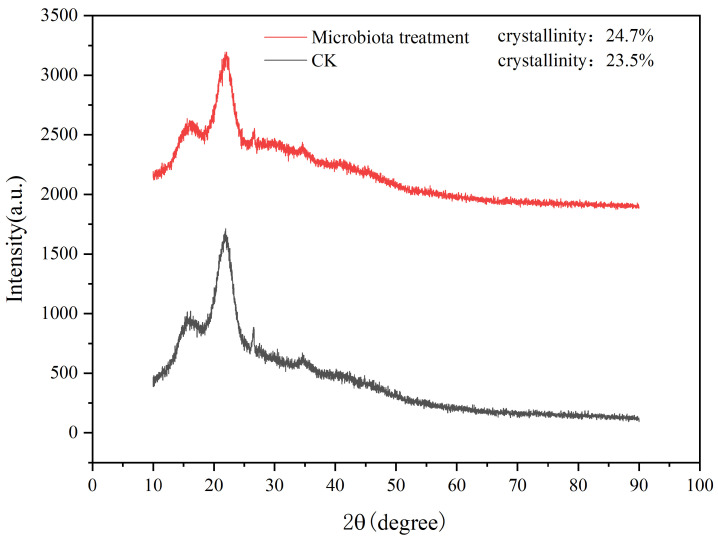
XRD diffraction analysis. CK represents the untreated straw; Microbiota treatment refers to the straw samples treated with the mixed actinomycetes flora.

Crystallinity serves as a crucial indicator for assessing the characteristics of cellulose, as it mirrors the extent of crystallization during the cellulose aggregation process [51]. Upon undergoing diverse pretreatments, with the elimination of specific constituents in the straw, alterations occur in both the structure and crystallinity of the straw. The X-ray diffraction (XRD) pattern, as depicted in Figure 9, exhibited main peaks and secondary peaks at 22.0° and 15.0°. These peaks, respectively, demonstrated the diffraction intensity of the crystalline and amorphous regions. Results indicated that after 7 days of fermentation treatment, the crystallinity of the straw control group (CK) was 23.5%, while the crystallinity of the experimental group (microbiota treatment) was 24.7%, exceeding that of the control group. In the course of biodegradation, the cellulase secreted eliminated a certain amount of lignin and a relatively larger quantity of hemicellulose components. This led to an elevation in the cellulose content and, consequently, a substantial augmentation in the overall crystallinity [52], which potentially accounted for this observed phenomenon. The data exhibited in Table 3, with a hemicellulose degradation rate of 64.98% and a lignin degradation rate of 65.85%, could potentially serve as evidence. They also imply that the supplementation of the microbial inoculant had a positive impact on the decomposition of straw.

### 3.4. Laboratory Simulation: Effect of Mixed Actinomycetes on Straw Returning Albic Soil

#### 3.4.1. Changes of Straw Weight Loss Rate in Different Treatment Samples

**Figure 10:**
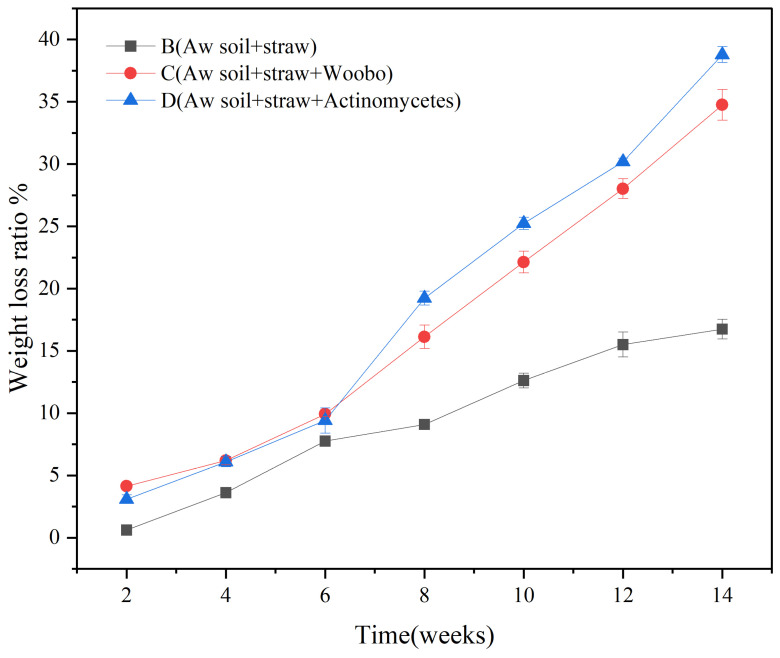
Changes of straw weight loss rate in different treatment samples. B, C, and D denote distinct experimental groups in the indoor simulated straw-returning experiment. B represents Aw soil with added straw, C represents Aw soil added with both straw and the commercial microbial agent Wooboo, and D represents Aw soil added with straw and mixed actinomycetes. Error bars are the standard error (n = 3).

For the purpose of more precisely assessing the straw degradation capacity of the compound actinomycetes, an experimental simulation of straw incorporation into albic soil was conducted within a laboratory setting. Three experimental groups and one blank control group were established. The dynamic alterations in the weight loss rate of corn straw are depicted in Figure 10. As the treatment duration was extended, the degradation rate of straw exhibited a remarkable augmentation. Commencing from the sixth week, the ascending tendency of the treatment in Group D was prominent, with Group C following. Nevertheless, during the period from the 10th to the 14th week, the upward trends of the degradation rates in both Group C and Group D decelerated. Initiating from the 14th week and persisting to the 16th week, the degradation efficiency once again manifested a conspicuous elevation. From an overall perspective, as time elapsed, the degradation rate of straw progressively increased. The comprehensive performance of Group D surpassed that of the addition of commercial microbial agents. In comparison with the control group, the degradation efficacy was substantially enhanced. The aforementioned experimental outcomes robustly indicate that the screened actinomycetes were capable of secreting a substantial quantity of highly efficient biodegradation enzymes. These enzymes actively engaged in the decomposition process of plant lignocellulose. Through their catalytic actions, they caused substantial disruption to the structure of macromolecular polysaccharides present within the straw. Additionally, the scanning electron microscopy (SEM) results, as illustrated in Figure 7C,D, provided conclusive evidence of the destructive impact of the actinomycete flora on the straw’s structure. It was also potentially correlated with the extensive environmental adaptability of actinomycetes since actinomycetes possess a certain degree of tolerance to some extreme environments (such as alkaline, high temperature or low temperature and other extreme environments) [53].

#### 3.4.2. Changes of Soil Organic Matter (SOM), Total Nitrogen (TN), Total Phosphorus (TP), TK, and pH in Different Samples

The addition of straw and exogenous microorganisms will bring about different changes to the chemical properties of the albic soil. In all samples (Table 5), SOM in the experimental group with special actinobacteria added (D6–D14) showed an increasing trend with the extension of culture time and reached 19.47 g/kg when the culture reached 14 weeks, which was the highest among all samples. Compared with the soil blank groups (A6–A14), the addition of straw increased SOM level as a whole, especially the addition of special actinomycetes significantly increased SOM content, indicating that the addition of actinomycetes promoted the decomposition of straw [54]. In all experimental groups, the alterations in total nitrogen (TN) and total phosphorus (TP) levels were not pronounced. This could potentially be attributed to a multitude of factors, including the specific nutrient release properties of straw and the intricate nature of the soil ecosystem. These aspects jointly contribute to the challenge of achieving significant changes in the contents of total nitrogen and total phosphorus within a short time frame [55]. The content of TK in blank control A6 was 7.84 mg/g, but decreased gradually with the extension of culture time, and reached 6.54 mg/g after 14 weeks of culture. The addition of straw (B6–B14) accelerated the decrease of TK content, showing an extremely significant trend. This might potentially be attributed to the incorporation of straw, which has served to stimulate the growth of soil microorganisms and expedite the metabolism of potassium (K) within the soil [56]; in addition, the addition of bacteriotics (C6–C14, D6–D14) gradually increased the total TK content and showed a gradual increasing trend with the culture time; compared with control group B, both showed a very significant trend. It may be due to the addition of exogenous microbial agents that the decomposition of straw was accelerated and the chemical components within the straw were released [57]. With the addition of straw and bactericide, the pH as a whole showed a weak acidity (5.06–5.17), and each component was not significant.

Based on the experimental results, it is evident that albic soil is characterized by poor soil nutrition, which poses an obstacle to crop growth [23]. Straw incorporation into the field proves to be an effective means to enhance the content of organic matter [23,58]. The supplementation of either commercial microbial agents (such as Woobao) or actinomycete flora led to alterations in soil chemical parameters. This is potentially attributable to the accelerated decomposition of straw. Particularly, the addition of actinomycete flora, as demonstrated by the data, exerted a notably positive impact on straw decomposition. Consequently, it can be concluded that the actinomycete flora holds definite application prospects and is applicable in the albic soil regions where straw is returned.

#### 3.4.3. OTUs Analysis in Different Experimental Groups

**Figure 11:**
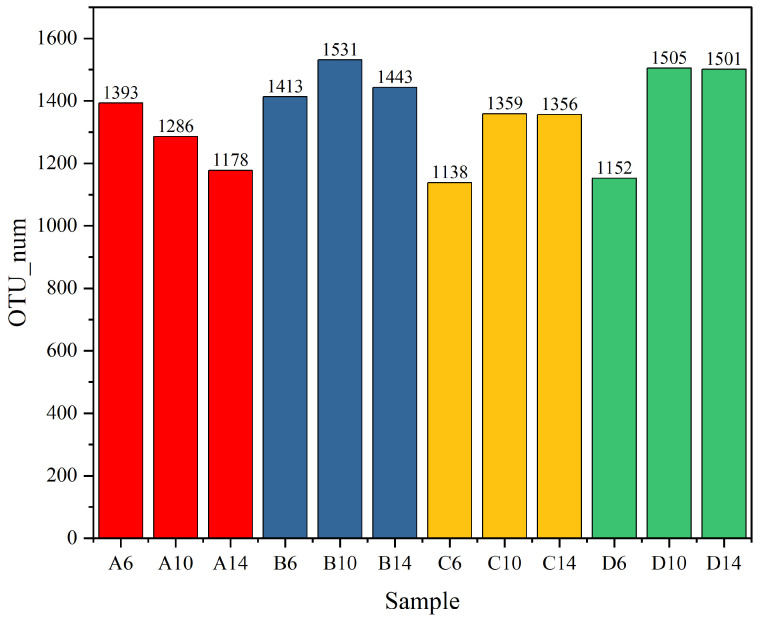
Different samples of OTU number of bacteria. In the indoor simulated straw-returning experiment, A, B, C, and D represent different experimental groups. A is Aw soil, B is Aw soil with added straw (both A and B are controls), C is Aw soil with straw and the commercial microbial agent Wooboo, and D is Aw soil with straw and mixed actinomycetes; 6, 10, and 14 represent different sampling times, and the unit is week.

As illustrated in the Figure 11, regarding the soil microorganisms sampled from the albic horizon soil, the bacterial abundance exhibited a gradual decline over the course of time. The quantity of OTUs diminished from 1393 in A6 to 1178 in A14. Subsequent to the incorporation of straw, the bacterial OTUs marginally augmented from 1413 in B6 to 1531 in B10. Nevertheless, by the 14th week (B14), a slight reduction was observed, yet it remained higher than that of A14, attaining 1443. This substantiates that the addition of straw can effectively enhance the soil species abundance [59]. In the experimental group C6 with the supplementation of the commercial microbial agent Woobao, the abundance was inferior to that of the soil in B6, amounting to 1138. This might be attributed to the introduction of dominant microbial communities, leading to nutrient competition. However, the abundance escalated by the 10th week (C10) and stabilized by the 14th week (C14), reaching 1365. Analogous to the experimental group with the addition of the commercial microbial agent, in group D with the addition of the specially prepared actinomycete flora, the bacterial abundances at corresponding time points were all greater than those in group C, with 1152 in D6 and remaining constant at 1501 from D10 to D14. It is particularly noteworthy that when the treatment reached 14 weeks, the species abundance in D14 was the pinnacle among all experimental groups.

**Figure 12:**
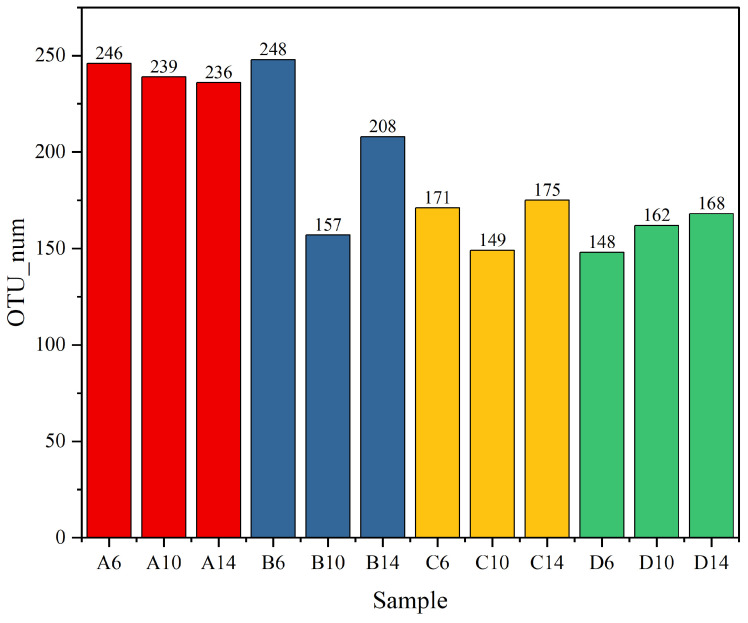
Different samples of OTU number of fungi. In the indoor simulated straw-returning experiment, A, B, C, and D represent different experimental groups. A is Aw soil, B is Aw soil with added straw (both A and B are controls), C is Aw soil with straw and the commercial microbial agent Wooboo, and D is Aw soil with straw and mixed actinomycetes; 6, 10, and 14 represent different sampling times, and the unit is week.

Concerning the fungal abundance (Figure 12), the OTU of the albic horizon soil sample did not manifest a significant alteration, fluctuating within the range of 236–246 in A6–A14. Following the addition of straw, the OUT data in B6 were not substantially disparate from that in A6. However, by the 10th week (B10), a pronounced decrease in abundance was witnessed, reaching 157. By the 14th week (B14), a minor elevation was detected, attaining 208. With the addition of the commercial microbial agent, in comparison with A6 and B6, the abundance was conspicuously diminished, reaching 171 in C6 and remaining stable at 175 by the 14th week (C14). Distinct from the other three groups, the addition of the specially prepared actinomycete flora (D6–D14) significantly curtailed the fungal abundance, remaining steady within the range of 148–168. Based on the comprehensive experimental outcomes, it can be deduced that the addition of the specially prepared actinomycete flora could effectively augment the bacterial abundance in the albic horizon of the albic soil with straw return, albeit the fungal abundance was moderately reduced. Compared with other types of soils, the albic horizon of albic soil had a lower microbial species abundance and a smaller number of active organisms [60,61].

#### 3.4.4. Analysis of Microbial Diversity in Different Experimental Groups

**Figure 13:**
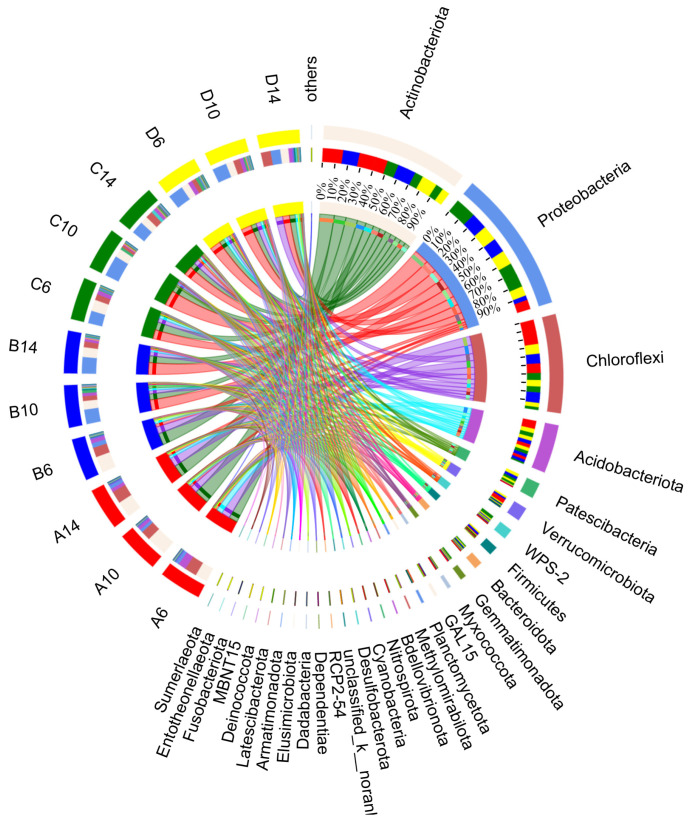
The sample and species relationship diagram, which shows the relative abundance and proportion of bacteria. A, B, C, and D correspond to the experimental groups of indoor simulated straw-returning settings, respectively; 6, 10, 14 represent different sampling times in weeks.

Circos was utilized to illustrate the relationship between samples and species. It can not only reflect the proportion of dominant species within each sample but also show the distribution of different dominant species among various samples. At the phylum level, significant differences in bacterial quantity were observed among different treatments (Figure 13). In the soil blank control groups (A6, A10, A14), *Actinobacteria* (with a proportion ranging from 31.51% to 52.39%) and *Chloroflexi* (from 24.51% to 30.95%) were the most abundant microorganisms, and the fluctuations with sampling time (6–14 weeks) were not obvious. After adding straw, notable changes occurred in the dominant microorganisms of the control groups (B6, B10, B14). In the samples collected in the sixth week, *Actinobacteria* (42.44%), *Chloroflexi* (24.74%) and *Proteobacteria* (11.73%) were predominant. In the 10th week samples, their abundances changed significantly. From the 10th to 14th week, the abundance of *Proteobacteria* increased to 37.31–42.25%, that of *Actinobacteria* decreased to 21.96–24.94%, and *Chloroflexi*’s abundance declined to 12.08–14.55%. The laboratory groups (C6, C10, C14) with the addition of the commercial microbial agent “Woobao” differed from the previous control groups. The dominant microbial groups were *Proteobacteria* (from 32.13% to 54.11%) and *Actinobacteria* (from 19.21% to 29.06%).

In the experimental groups (D6, D10, D14) with a special *Actinobacteria* flora, compared to the blank control groups (A6, A10, A14), the abundance of *Proteobacteria* increased remarkably (from 24.76% to 39.18%). *Proteobacteria* is a eutrophic bacterium [62] and has a large proportion in soil community composition and relative abundance [8], while the abundance of *Actinobacteria* remained stable at 18.94–20.94%. As a Gram-positive bacterium, *Actinobacteria* is crucial for organic matter turnover, including cellulose and chitin decomposition [63]. The results of the straw weight loss rate (Figure 10) also confirmed *Actinobacteria*’s significant contribution to straw decomposition. Starting from the sixth week, the straw weight loss rate rose sharply and was the highest among all experimental groups, which further proved that the specially prepared mixed *Actinobacteria* flora had a positive promoting effect on straw decomposition. The abundance of *Acidobacteria* was stable at 8.93–11.12%. Most *Acidobacteria* are acidophilic, and their abundance is inversely related to soil pH. In a soil environment with a relatively low pH, the abundance of *Acidobacteria* reaches its maximum [64], which is verified by the pH results in Table 5. With the extension of incubation time, the abundance of *Firmicutes* increased significantly.

**Figure 14:**
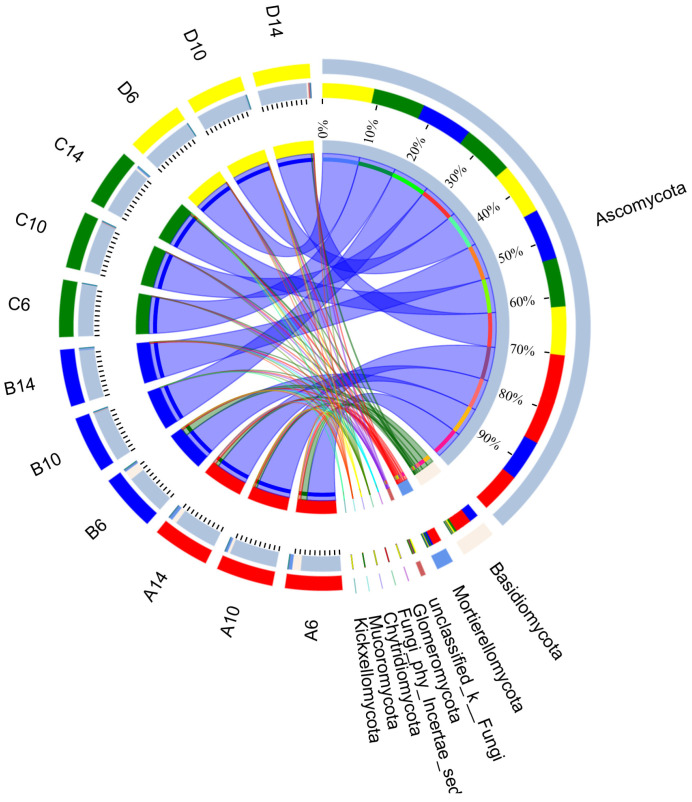
The sample and species relationship diagram, which shows the relative abundance and proportion of Fungi. A, B, C, and D correspond to the experimental groups of indoor simulated straw-returning settings, respectively; 6, 10, and 14 represent different sampling times in weeks.

At the phylum level, notable disparities in the fungal quantity were also manifested among diverse treatment modalities (Figure 14). In the soil blank control groups (A6, A10, A14), the microorganisms exhibiting the highest abundances were *Ascomycota* (with the proportion spanning from 76.13% to 90.01%), *Basidiomycota* (ranging from 5.40% to 16.35%), and *Mortierellomycota* (ranging from 4.16% to 6.66%). Certain species within *Mortierellomycota* are significant saprophytic fungi, being capable of decomposing complex organic matter in the soil, such as cellulose and chitin, among others [65]. With the prolongation of the sampling period, no conspicuous alterations were discerned in the soil blank control groups. In contrast, in the soil samples with the addition of straw, the relative abundance progressively declined from the peak value in the sixth week. This could be attributed to the introduction of a substantial amount of straw carbon source, thereby giving rise to nutritional competition between the dominant cellulose-degrading microbial populations and the microorganisms of this phylum. Among all the experimental groups, with the exception of the B6 group with the addition of straw, whose abundance was relatively proximate to that of the A6 group in the soil blank group, no evident changes were observable in the other experimental groups. They all took *Ascomycota* as the dominant microorganism, and its abundance was above 92%.

During the exploration of the influence of time on species abundance, a significant negative correlation between pH value and species abundance was manifested [66]. As presented in Table 1, at the D14 time point within the experimental group, the measured pH value was 5.06, which was marginally lower than the 5.16 recorded at C14 of the same experimental cohort. Concurrently, the quantity of operational taxonomic units (OTUs), serving as an indicator of bacterial species abundance, attained 1501 at D14 of the experimental group, exceeding the 1356 OTUs at C14. In the control group A14, the pH value was 5.52, accompanied by a corresponding OTU number of 1178. Similarly, the variation trend of fungal abundance was in accordance with that of bacteria [67]. Moreover, the total nitrogen content exhibited a positive correlation with the pH value. This observed phenomenon is congruent with the outcomes of other research studies [68,69].

## 4. Conclusions

### 4.1. Characteristics of Albic Soil and Straw Returning in Farm 852

Albic soil, with its poor nutrition, impedes crop growth and undermines national food security and yield-boosting strategies. Straw returning is a viable solution, but in Northeast China, low temperatures, limited effective accumulated temperature, and scarce microbial abundance slow down straw decomposition. Statistically, the annual degradation rate is only 45–60%, and the three-year cumulative rate is 80–90%. Experimental results showed that the albic soil in Farm 852 was nutritionally poor. Within the soil profile from the surface layer down to a depth ranging from 0 to 40 cm, the nutrient content exhibited a progressive decline. Soil organic matter (SOM) dropped from 36.13 g/kg to 9.43 g/kg, total nitrogen (TN) from 1.88 g/100 g to 0.69 g/100 g, total phosphorus (TP) from 0.73 mg/g to 0.29 mg/g, and total potassium (TK) from 18.71 mg/g to 17.36 mg/g. Conversely, the soil pH rose slightly from 4.77 to 5.03.

### 4.2. Screening of Actinomycetes and Construction of Microbial Community

This study isolated 10 excellent straw-degrading strains from Farm 852’s albic soil, including a newly identified strain *Nonomuraea* sp. CP82^T^, which were well suited to the local soil environment. Another new strain, *Streptomyces* sp. NC5^T^, sourced from other soil samples, also showed strong straw-degrading potential. A composite strain consortium of mixed actinomycetes, incorporating *Streptomyces* sp. NC5^T^, *Nonomuraea* sp. CP82^T^, *Streptomyces* sp. JD3, and *Pseudomonas* sp. MD68, was successfully constructed. After 7 days of fermentation, it achieved remarkable degradation rates: 69.07% for cellulose, 64.98% for hemicellulose, and 68.95% for lignin, outperforming single strains. FTIR, XRD, and SEM analyses confirmed its structural damage to straw, indicating its potential to hasten decomposition.

### 4.3. Effect of Actinomycete Flora in Albic Soil with Straw Returning in Laboratory Simulation Experiments

Laboratory simulations of straw returning to albic soil further validated the consortium’s capabilities and its impact on soil. Against commercial microbial agents as a benchmark, adding the mixed actinomycetes proved more effective in degrading straw, with a peak 16-week straw weight loss rate of 38.78%. After 14 weeks, the D14 group with mixed actinomycetes significantly enhanced soil microbial abundance. The bacterial OTUs hit 1501, the highest among groups, while fungal OTUs were 168, the lowest, suggesting bacteria as the main drivers of straw degradation. Compared to the B14 group, Group D maximized the soil organic matter (SOM) content at 19.47 g/kg. Although TN and TK levels showed no clear trends, the TP content in Group C and D was generally higher than the control, likely due to exogenous microbes accelerating straw component release.

In conclusion, the mixed actinomycete consortium holds great promise for improving straw decomposition and soil quality in albic soil regions, offering a practical approach to agricultural waste management and soil remediation.

## Figures and Tables

**Table 1:** Characteristics of albic soil in Farm 852.

Sample	SOM (g/kg)	TN (g/100 g)	TP (mg/g)	TK (mg/g)	pH
Ap-H (0–10)	36.13 ± 0.12 a	1.88 ± 0.02 a	0.73 ± 0.01 a	18.71 ± 0.21 a	4.77
Ap-L (10–20)	34.22 ± 0.21 a	1.95 ± 0.01 a	0.61 ± 0.00 b	18.64 ± 0.09 a	4.64
Aw-H (20–30)	15.32 ± 0.07 b	1.01 ± 0.00 b	0.35 ± 0.00 c	16.20 ± 0.26 b	5.38
Aw-L (30–40)	9.43 ± 0.13 c	0.69 ± 0.01 c	0.29 ± 0.00 d	17.36 ± 0.12 a	5.03

“H” and “L” indicate the upper and lower regions of the layer; the numbers represent depth of soil layer, cm. The values are presented as mean ± standard error (n = 3). Different letters signify significant differences among treatments (*p* < 0.05).

**Table 2:** Disintegration experiment of strain filter strip.

Strain	Disintegration of Filter Paper
CP82	+++
MD7	+
MDB7	+
MD15	+++
MD31	+++
SY6	++
MD43	++
MD44	+
MD50	+++
MD53	++
MD55	+++
MD28	+
MD63	+++
MD68	+++
JD30	+++
NC5	+++
JSTG24	+++
GS9	+++

Strains capable of reducing the filter paper to a pulpy consistency were designated as “+++”. Strains that exhibited a certain degree of degradation effect on the filter paper were labeled as “++”, while those that caused no obvious damage to the filter paper were marked as “+”.

**Table 3:** Degradation effects of different strains on the main components of straw.

	Cellulose (%)	Hemicellulose (%)	Lignin (%)
MD68	61.01 ± 1.29 c	54.54 ± 1.01 b	59.88 ± 2.31 c
NC5	62.97 ± 2.03 b	38.63 ± 2.11	42.09 ± 1.41
MD55	53.20 ± 0.79	46.60 ± 3.12	50.35 ± 1.67 d
MD63	63.40 ± 1.01 b	31.58 ± 1.29	44.88 ± 1.91
GS9	57.15 ± 0.81	31.07 ± 1.33	47.56 ± 2.22
MD31	45.82 ± 1.11	48.35 ± 2.07 c	40.00 ± 1.53
JD30	52.75 ± 1.72	30.00 ± 0.62	44.19 ± 1.01
JSTG24	26.15 ± 0.21	55.36 ± 1.81 b	71.16 ± 1.35 a
MD15	45.16 ± 1.91	28.87 ± 2.37	45.58 ± 1.87
CP82	44.58 ± 1.37	28.08 ± 1.19	22.33 ± 1.44
Mixed actinomycetes	69.07 ± 1.98 a	64.98 ± 2.11 a	68.95 ± 1.58 b

The values are presented as mean ± standard error (n = 3). Different letters signify significant differences among treatments (*p* < 0.05). Among them, the significance difference was marked only for the four groups of data with the highest values for each indicator.

**Table 4:** Sequencing results of active strains.

Strain	Similar Strains	Label of Similar Strains	Similarity Degree	Taxonomic Classification Hierarchy
CP82	*Nonomuraea zeae*	DSM 100528	99.72%	*Bacteria*; *Actinobacteria*; *Actinomycetia*; *Streptosporangiales*; *Streptosporangiaceae*; *Nonomuraea*
JD30	*Streptomyces broussonetiae*	T44	99.24%	*Bacteria*; *Actinobacteria*; *Actinomycetia*; *Streptomycetaes*; *Streptomycetaceae*; *Steptomyces*
MD31	*Streptomvces canus*	DSM 40017	99.64%	
MD55	*Streptomyces fagopyri*	QMT-28	98.77%	
MD63	*Streptomyces cinnabarigriseus*	JS360	99.28%	
MD15	*Streptomyces phaeochromogenes*	NBRC 3180	99.28%	
JNC5	*Streptomvces malavsiense*	MUSC 136	99.42%	
JSTG24	*Streptomvces barringtoniae*	JA03	98.90%	
GS9	*Streptomyces rishiriensis*	NBRC 13407	99.01%	
MD68	*Pseudomonas migulae*	CIP 105470	99.57%	*Bacteria*; *Proteobacteria*; *Gammaproteobacteria*; *Pseudomonadaies*; *Pseudomonadaceae*; *Pseudomonas*

**Table 5:** Changes of soil organic matter (SOM), TN, TP, TK and pH in different samples.

Sample	SOM (g/kg)	TN (g/100 g)	TP (mg/g)	TK (mg/g)	pH
A6	11.62 ± 0.22 c	0.18 ± 0.00 b	0.35 ± 0.01 b	7.84 ± 0.30 a	5.37
B6	14.22 ± 0.13 a	0.20 ± 0.01 a	0.37 ± 0.00 a	6.54 ± 0.09 c	5.17
C6	14.05 ± 0.09 a	0.19 ± 0.01 a	0.39 ± 0.01 a	6.52 ± 0.25 d	5.08
D6	13.80 ± 0.02 b	0.20 ± 0.01 a	0.39 ± 0.01 a	7.14 ± 0.07 b	5.12
A10	11.88 ± 0.41 b	0.17 ± 0.00 b	0.35 ± 0.01 b	6.86 ± 0.06 a	5.52
B10	14.10 ± 0.29 a	0.20 ± 0.01 a	0.38 ± 0.01 a	6.85 ± 0.26 a	5.13
C10	13.86 ± 0.30 a	0.21 ± 0.01 a	0.39 ± 0.01 a	6.52 ± 0.07 b	5.15
D10	13.46 ± 0.18 a	0.20 ± 0.01 a	0.36 ± 0.00 b	7.31 ± 0.26 a	5.10
A14	11.55 ± 0.24 d	0.19 ± 0.00 b	0.34 ± 0.01 b	6.51 ± 0.21 b	5.52
B14	13.42 ± 0.20 c	0.21 ± 0.00 a	0.38 ± 0.00 a	6.05 ± 0.08 c	5.15
C14	14.53 ± 0.15 b	0.21 ± 0.00 a	0.38 ± 0.02 a	7.38 ± 0.06 a	5.16
D14	19.47 ± 0.15 a	0.19 ± 0.01 b	0.36 ± 0.01 b	7.18 ± 0.12 a	5.06

In the indoor simulated straw-returning experiment, A, B, C, and D denote different experimental groups. A is Aw soil, and B is Aw soil with added straw (both A and B serve as controls). C is Aw soil with straw and the commercial microbial agent Wooboo, while D is Aw soil with straw and mixed actinomycetes; 6, 10, and 14 represent sampling times in weeks. The values are presented as mean ± standard error (n = 3). Different letters signify significant differences among treatments (*p* < 0.05).

## Data Availability

The original contributions presented in this study are included in the article/Supplementary Material. Further inquiries can be directed to the corresponding author.

## Supplementary Materials

The following supporting information can be downloaded at: https://www.mdpi.com/article/10.3390/microorganisms13020385/s1, Figure S1: *Streptomyces* sp. NC5^T^ colonization culture in albic soil; Figure S2: Disintegrating ability of filter paper strip; Figure S3: The phylogenetic tree (A) and colony morphology (B) of the *Nonomuraea* sp. CP82^T^; Table S1: The ratio of transparent circle and colony diameter of different active strains; Table S2: Results of the confrontation test of 11 strains.
