# Supplementary material for: Construction and Effect Analysis of a Mixed Actinomycete Flora for Straw Returning to Albic Soil in Northeast China
Source: Microorganisms. 2025 Feb 10;13(2):385. doi: 10.3390/microorganisms13020385 (PMC11858276; doi:10.3390/microorganisms13020385)
Supplement: Supplementary file 1 [file microorganisms-13-00385-s001.zip › Figure S1 Streptomyces sp. NC5T colonization culture in albic soil..pdf]

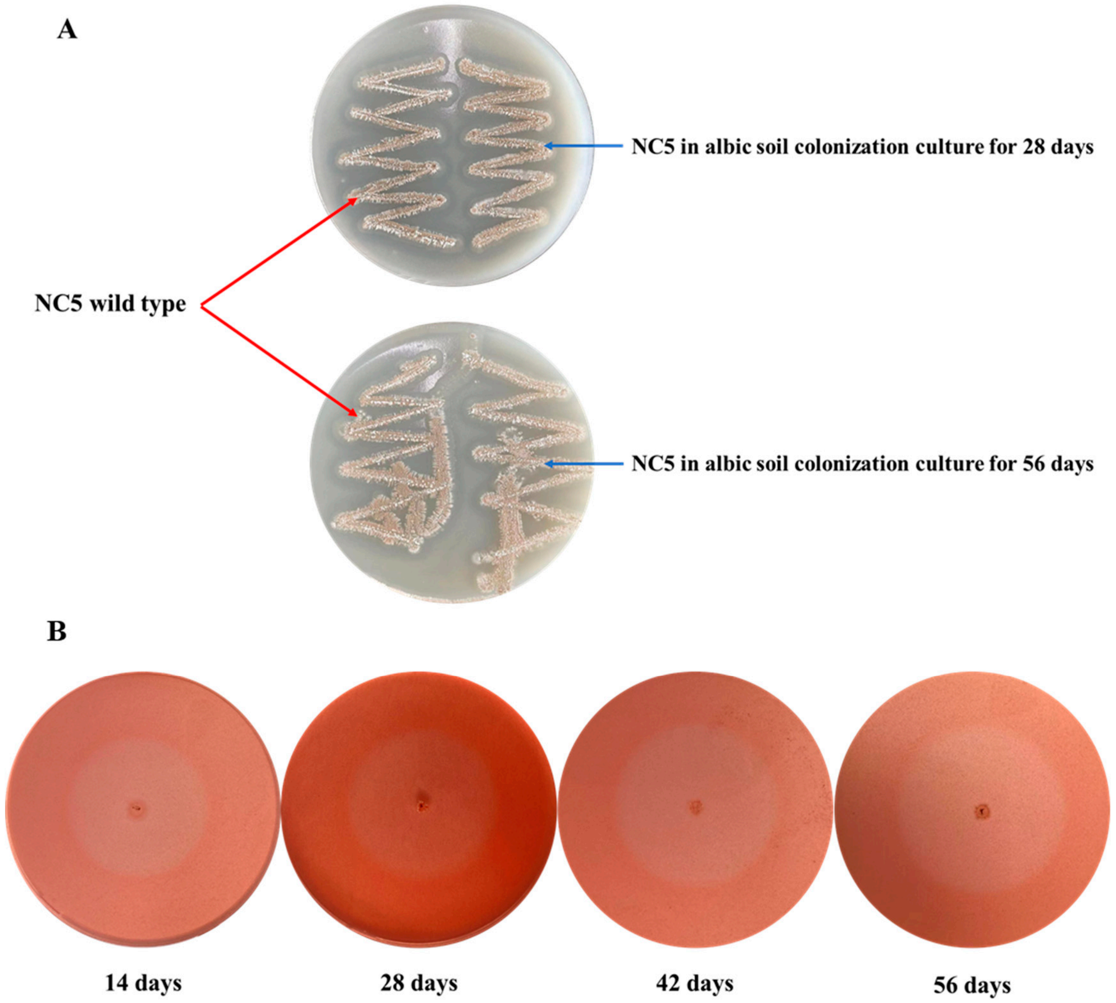

Figure S2. *Streptomyces sp.* NC5^T colonization culture in albic soil.

The albic soil colonization experiment was carried out with the aim of observing the adaptability of the strain originating from its corresponding soil source to the albic soil environment. According to the experimental result Figure S1A, following a cultivation period of 28 days and 56 days, it was evident that the strain could adapt favorably to the albic soil environment and exhibited normal growth. As depicted in Figure S1B, the ratio of Congo red staining to colony diameter of NC5 subsequent to albic soil culturing did not display a pronounced changing trend. This substantiates that the strain is well-suited for the albic soil environment and thus can be considered as an outstanding candidate strain for further experimental investigations.
