# Supplementary material for: Construction and Effect Analysis of a Mixed Actinomycete Flora for Straw Returning to Albic Soil in Northeast China
Source: Microorganisms. 2025 Feb 10;13(2):385. doi: 10.3390/microorganisms13020385 (PMC11858276; doi:10.3390/microorganisms13020385)
Supplement: Supplementary file 1 [file microorganisms-13-00385-s001.zip › Table S1. The ratio of transparent circle and colony diameter of different active strains.pdf]

Table S1. The ratio of transparent circle and colony diameter of different active strains

Active Strain	The Ratio of the Transparent Circle to the Colony Diameter
A31	4.28
A35	2.16
A36	3.52
A37	3.87
A43	5.06
A44	4.15
A45	3.5
A63	6.72
A64	3.26
A65	2.98
A67	3.43
A68	4.75
A47	3.79
50	4.23
B38	2.76
A32	3.16
A50	3.57
C28	3.69
A44	3.32
B7	3.91
B36	2.87
CP61	0.31
GS62	3.31
GS24	6.35
6	4.19
72	4.3
9	3.85
39	3.48
24	4.25
83	4.61
82	5.03
JD30	4.33
CP82	4.06
MD7	4.36
MD31	7.03
SY6	3.67
MD43	5.45
MD55	4.52
MD59	4.66
MD63	6.95
MDB7	4.34

MD15	4.33
MD28	5.57
JSTG24	3.93
MD44	5.38
MD50	5.78
MD53	4.83
MD68	7.69
SY57	4.92
SY72	5.11
GS9	9.75
MR1	1.71
MR2	2.68
MR5	1.5
MR6	2.43
MR7	2.75
MR8	1.83
MR9	1.92
MR16	3.11
MR24	3.26
MR37	3.45
MR40	2.87
MR74	2.36
MR75	2.98
MR80	2.77
MR91	2.69
MR102	3.12
MR96	3.49
MR100	3.78
MR102	2.82
MR138	3.53
MR143	3.2
MR152	3.74
MR155	3.86
MR158	2.57
MR160	3.69
MR171	2.81
MR172	2.94
MR177	3.06
MR178	2.97
MR187	2.86
MR188	3.23
MR191	2.88
MR192	3.16
