# Supplementary material for: Construction and Effect Analysis of a Mixed Actinomycete Flora for Straw Returning to Albic Soil in Northeast China
Source: Microorganisms. 2025 Feb 10;13(2):385. doi: 10.3390/microorganisms13020385 (PMC11858276; doi:10.3390/microorganisms13020385)
Supplement: Supplementary file 1 [file microorganisms-13-00385-s001.zip › Table S2. Results of the confrontation test of 11 strains.pdf]

Table S2. Results of the confrontation test of 11 strains

	JD30	MD15	MD31	MD50	MD55	MD63	MD68	CP82	NC5	JSTG24	GS9
**JD30**		×	×	√	√	√	√	√	√	√	√
**MD15**	×		√	√	√	×	√	√	√	√	×
**MD31**	×	√		√	√	√	√	√	√	√	√
**MD50**	√	√	√		√	×	√	√	√	√	√
**MD55**	√	√	√	√		√	√	√	×	√	√
**MD63**	√	×	√	×	√		√	√	√	×	√
**MD68**	√	√	√	√	√	√		√	√	√	√
**CP82**	√	√	√	√	√	√	√		√	√	√
**NC5**	√	√	√	√	×	√	√	√		×	√
**JSTG24**	√	√	√	√	√	×	√	√	×		√
**GS9**	√	×	√	√	√	√	√	√	√	√	

"√" indicates that the strains can grow together, while "×" indicates the presence of antagonistic effects and the inability to coexist.
