# Supplementary figures and images for: Construction and Effect Analysis of a Mixed Actinomycete Flora for Straw Returning to Albic Soil in Northeast China
Source: Microorganisms. 2025 Feb 10;13(2):385. doi: 10.3390/microorganisms13020385 (PMC11858276; doi:10.3390/microorganisms13020385)

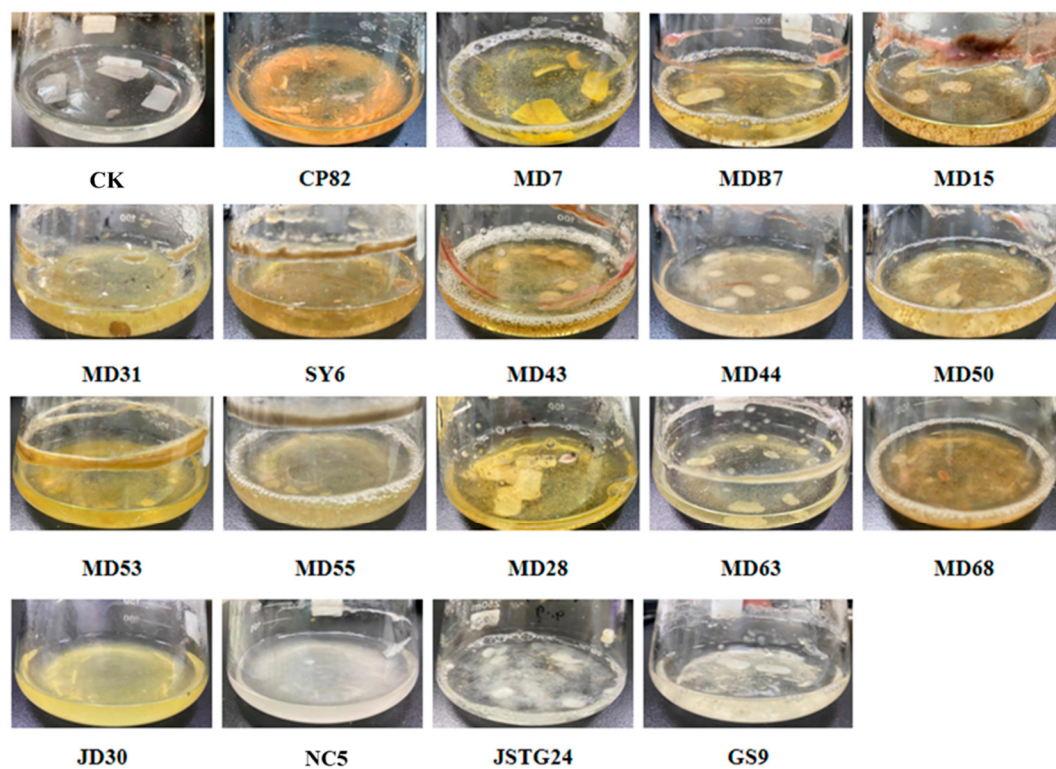

Figure S2. Disintegrating ability of filter paper strip.

Supplement: Supplementary file 1 [file microorganisms-13-00385-s001.zip › Figure S2 Disintegrating ability of filter paper strip.pdf]

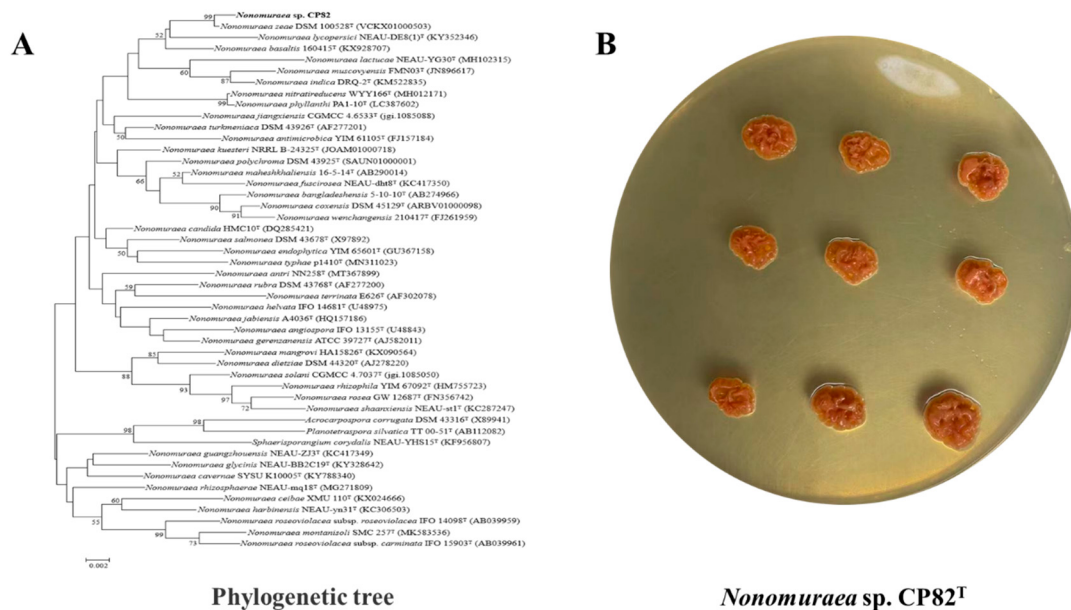

Figure S3. The phylogenetic tree (A) and colony morphology (B) of the *Nonomuraea* sp. CP82<sup>T</sup>

Supplement: Supplementary file 1 [file microorganisms-13-00385-s001.zip › Figure S3. The phylogenetic tree (A) and colony morphology (B) of the Nonomuraea sp. CP82T.pdf]
